# The efficacy and safety of botulinum toxin type A in treatment of trigeminal neuralgia and peripheral neuropathic pain: A meta‐analysis of randomized controlled trials

**DOI:** 10.1002/brb3.1409

**Published:** 2019-09-21

**Authors:** Jiangshan Wei, Xiangyu Zhu, Guang Yang, Jun Shen, Peng Xie, Xiaohua Zuo, Lei Xia, Qiu Han, Ying Zhao

**Affiliations:** ^1^ Department of Neurology Hongze Huai'an District People's Hospital Huai'an China; ^2^ ICU The Second People's Hospital of Huai'an Huai'an Affiliated Hospital of Xuzhou Medical University Huai'an China; ^3^ Department of Neurology The Second People's Hospital of Huai'an The Affiliated Huai'an Hospital of Xuzhou Medical University Huai'an China; ^4^ Department of Neurosurgery The Second People's Hospital of Huai'an The Affiliated Huai'an Hospital of Xuzhou Medical University Huai'an China; ^5^ Department of Pain Management The Second People's Hospital of Huai'an The Affiliated Huai'an Hospital of Xuzhou Medical University Huai'an China; ^6^ Department of Neurology Huai'an First People's Hospital The Affiliated Huai'an No. 1 People's Hospital of Nanjing Medical University Huai'an China

**Keywords:** botulinum toxin‐A, meta‐analysis, peripheral neuropathic pain, randomized controlled trials, trigeminal neuralgia

## Abstract

**Background:**

Although recent studies have shown that botulinum toxin‐A (BTX‐A) has a good analgesic effect on trigeminal neuralgia (TN) and peripheral neuropathic pain (PNP), the quality of evidence is low due to limited data. This meta‐analysis is used to synthesize existing evidence for the treatment of these conditions with BTX‐A.

**Methods:**

Relevant trials were accessed by using an electronic search in databases (Web of Science, PubMed, EMBASE, Cochrane Library, and http://ClinicalTrials.gov). Data from included randomized controlled trials (RCTs) on the efficacy and safety of BTX‐A in treating TN and PNP were extracted for meta‐analysis.

**Results:**

Finally, 10 RCTs (*n* = 391) were included in this meta‐analysis. The pooled effect of BTX‐A was superior to placebo based on pain intensity (SMD = −0.48, 95% CI [−0.74, 0.23] at 1 month, SMD = −0.58, 95% CI [−0.91, −0.24] at 2 months, and SMD = −0.55, 95% CI [−0.87, −0.22] at 3 months). Number needed to treat (NNT) for 50% pain intensity reduction showed better effect of BTX‐A on TN and postherpetic neuralgia (PN). Adverse events associated with BTX‐A were similar to placebo (OR = 1.58, 95% CI [0.51, 4.87], *p* = .424).

**Conclusion:**

Pooled data from our meta‐analysis suggest that BTX‐A is efficacious and safe in treating TN and PNP. However, due to the limited sample size and heterogeneity, further larger and well‐designed RCTs are imperative to validate these findings.

## Graphical abstract

In summary, our pooled results support the injection of botulinum toxin‐A as a promising alternative treatment for trigeminal neuralgia and peripheral neuropathic pain. Further larger and well‐designed randomized controlled trials are still needed to provide more in‐depth insight into current issues.

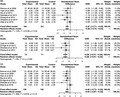

## 1 INTRODUCTION

Neuropathic pain is defined as pain caused by a lesion or disease of the somatosensory system and affects 7%–10% of the general population (Colloca et al., 2017; Finnerup et al., 2016). Peripheral neuropathic pain (PNP) is the most common type of neuropathic pain presenting in the clinical conditions. As the population ages, its incidence will continue to increase (Colloca et al., 2017). Neuropathic pain, as a largely unmet medical need (Finnerup et al., 2015), is one of the most difficult pain syndromes to manage, and outcomes often are unsatisfactory (van Hecke, Austin, Khan, Smith, & Torrance, 2014). It seriously affects the quality of life of patients owing to increased drug prescriptions and visits to healthcare providers, as well as the morbidity from the pain itself and the inciting disease (Colloca et al., 2017). It imposes a huge economic burden on the individual and society (Attal, Lanteri‐Minet, Laurent, Fermanian, & Bouhassira, 2011; Doth, Hansson, Jensen, & Taylor, 2010; Langley, Van Litsenburg, Cappelleri, & Carroll, 2013).

Medication and neurosurgery are two major therapies for neuropathic pain. These currently recommended first‐line medical treatments may only partially relieve pain in 30%–40% of patients (Hansson, Attal, Baron, & Cruccu, 2009) and are accompanied by systemic adverse events (Freynhagen et al., 2015). Surgical interventions carry a risk of serious and intractable complications and even worsen the initial conditions.

Botulinum toxin‐A (BTX‐A) is a potent neurotoxin produced from *Clostridium botulinum* strains (Oguma, Fujinaga, & Inoue, 1995). It can inhibit release of acetylcholine from neuromuscular junctions, causing muscle relaxation (Humeau, Doussau, Grant, & Poulain, 2000; Pearce, First, MacCallum, & Gupta, 1997). Experiment studies demonstrated that BTX‐A affects the presynaptic vesicles of neurons by inhibiting the release of certain neurotransmitters such as acetylcholine and the nociceptive neuropeptides, substance P, calcitonin gene‐related peptide, and glutamate (Jeynes & Gauci, 2008; Lakhan, Velasco, & Tepper, 2015). It also inhibits the expression of vanilloid receptor TRPV1 on the surface of peripheral nociceptors that are responsible for inflammatory hyperalgesia (Aoki, 2008; Jeynes & Gauci, 2008; Lakhan et al., 2015). Furthermore, studies have indicated that analgesic action of BTX‐A is independent of its relaxation of the muscle (Dolly & O'Connell, 2012). Recent randomized controlled trials (RCTs) had also provided evidence that BTX‐A is effective on treating chronic pain conditions such as trigeminal neuralgia (TN), PN, diabetic neuropathic pain (DNP), post‐traumatic neuralgia, or chronic migraine (Dodick et al., 2010; Ranoux, Attal, Morain, & Bouhassira, 2008; Shehata, El‐Tamawy, Shalaby, & Ramzy, 2013; Xiao et al., 2010; Yuan et al., 2009; Zhang et al., 2014; Zuniga, Piedimonte, Diaz, & Micheli, 2013).

However, it is lack of power to elucidate the efficacy and safety implications of BTX‐A for TN and PNP owing to small sample size. Meta‐analysis method can pool the data from small studies to provide evidence with better power. Hence, we performed this meta‐analysis to synthesize all this evidence from the published RCTs on the efficacy and safety of BTX‐A in treating TN and PNP.

## 2 METHODS

### 2.1 Search strategy

We have confirmed that our study follows the recommendation of Preferred Reporting Items for Systematic Reviews and Meta‐Analyses (PRISMA) statement (Liberati et al., 2009). We searched in the electronic databases (Web of Science, PubMed, EMBASE, Cochrane Library, and http://ClinicalTrials.gov) from 1988 to May 2018. The MeSH headings were as follows: “Neuralgia,” “Trigeminal Neuralgia,” “Peripheral Nervous System Diseases,” “Diabetic Neuropathies,” “Neuralgia, Postherpetic,” “Facial Neuralgia,” “Sciatica,” “Sciatic Neuropathy,” “Neuritis,” “Brachial Plexus Neuritis,” “Median Neuropathy,” “Botulinum Toxins.” Citations of included publications were identified additionally. Individual was restricted to humans, and language was restricted to English. Moreover, we searched for unpublished gray literature in http://ClinicalTrials.gov.

### 2.2 Study selection

We imported search results from the aforementioned electronic databases into EndNote X6 (Thompson Reuter) for selection. Two authors independently screened the references according to the predetermined inclusion and exclusion criteria. The inclusion criteria were as follows: (a) RCTs evaluating efficacy and safety of BTX‐A on TN and PNP; (b) no limitation to age of patients and study area. The exclusion criteria were as follows: (a) unreliably extracted data; (b) datasets from articles unable to extract; and (c) case reports, case series, reviews, commentaries, errata, notes, letters, and abstract articles only. All conflicts in screening step were discussed between two authors to reach an agreement, and supervisor adjudicated unresolved conflicts if necessary. The full texts of all included trials were downloaded for screening to identify available data for extraction.

### 2.3 Outcome measures

Outcomes measures as follow were used to evaluate the efficacy and safety of BTX‐A in treating TN and PNP: (a) visual analog scale (VAS) score, neuropathy pain scale (NPS), and numeric rating scale (NRS) score; (b) NNT (with pain score reduced >50%); and (c) adverse events associated with BTX‐A injections.

### 2.4 Data extraction and quality assessment

The standardized template was developed for extraction. The relevant data were extracted into the template independently by two authors. Extracted data included: journal, author, publication year, sample size, population, patients' gender and age, injection route, duration of treatment, study design, follow‐up time, and main outcomes measures. Cochrane risk‐of‐bias tool was used to assess the methodological quality of the included trials based on sequence generation, allocation concealment, blinding, incomplete outcome data, selective outcome reporting, and other sources of bias (Higgins & Green, 2008).

### 2.5 Data synthesis

Statistical analysis was carried out with R3.4.3 (http://www.r-project.org/). Continuous variables were pooled as standard mean difference (SMD) using the generic inverse variance method, while dichotomous data were aggregated as odds ratio (OR) by Mantel–Haenszel (M‐H) method. All analyses were conducted using random‐effect and fixed‐effect model. The sensitivity analysis was used to assess the effect of the hypothetical model on the overall effect size, by comparing the analysis between the random‐effect model and fixed‐effect model. Heterogeneity analysis was performed with *I*‐square and chi‐square tests across the included trials. *p*‐value < .05 was identified as a statistically significant level.

## 3 RESULTS

### 3.1 Search results and characteristics of eligible RCTs

**Figure 1:**
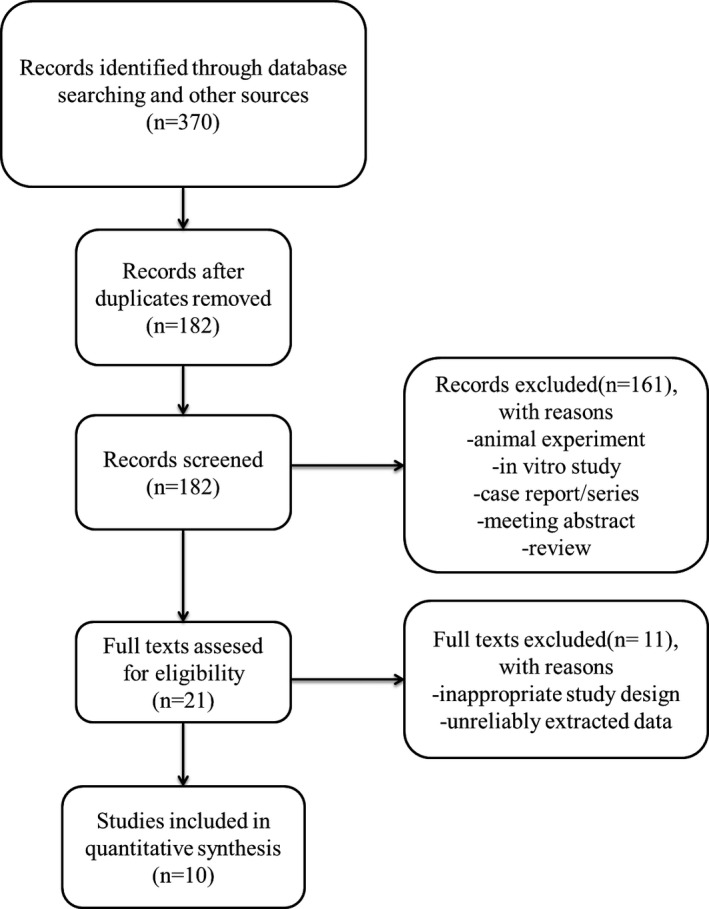
PRISMA flow diagram

The detailed characteristics of eligible RCTs are summarized in Table 1. A total of 391 patients from 10 trials were included in this analysis (Figure 1). From 10 RCTs (Apalla, Sotiriou, Lallas, Lazaridou, & Ioannides, 2013; Attal et al., 2016; Ghasemi, Ansari, Basiri, & Shaigannejad, 2014; Ranoux et al., 2008; Shehata et al., 2013; Wu et al., 2012; Xiao et al., 2010; Yuan et al., 2009; Zhang et al., 2014; Zuniga et al., 2013), the mean age of BTX‐A group ranged from 51.6 to 73.2, and mean age ranged from 49.7 to 77.5 for placebo group. There was a statistically insignificant difference in baseline pain intensity between the BTX‐A and placebo groups in all included trials. The duration of follow‐up was ranging from 8 to 24 weeks. The administration route, the injection site, the dosage of BTX‐A injected, and the injection number varied among included trials. The dosage of BTX‐A injections ranged from 25 U in study of Zhang et al. (2014) to 300 U in study of Attal et al. (2016). Routes for BTX‐A injection include subcutaneous, submucosal, or intradermal.

**Table 1:** Basic characteristics of eligible RCTs

Reference	Basic characteristic: age; M/F (*n*); Country	Condition	Design	BTX‐A: dose U, route, numbers of injections	Outcome measure
Attal et al. (2016)	BTX‐A: 51.6 (16.7); 17/17 (34) Placebo: 52.3 (15.8); 20/12 (32) France, Brazil	PNP	RCT, double blind	Up to 300 U, subcutaneous, two times (several injections)	NRS AE
Apalla et al. (2013)	BTX‐A: 73.2 (10.5); 8/7 (15) Placebo: 77.5 (8.2); 10/5 (15) Greece	PN	RCT, double blind	100 U, subcutaneous, 40 injections	VAS
Ghasemi et al. (2014)	BTX‐A: 62.7 (9.9); 13/7 (20) Placebo: 59.3 (9.6); 9/11 (20) Iran	DNP	RCT, double blind	100 U per each foot, intradermal, 12 injections	NPS AE
Ranoux et al. (2008)	BTX‐A: 53.8 (13.9); 6/9 (15) Placebo: 49.7 (15.9); 4/10 (14) France	PN	RCT, double blind	20−190 U, intradermal, <40 injections	VAS AE
Shehata et al. (2013)	Total: 45.95 (10.02); 9/11 (20) Egypt	TN	RCT, single blind	40−60 U, subcutaneous, 8–12 injections	VAS
Wu et al. (2012)	BTX‐A: 59.14 (12.58); 9/13 (22) Placebo: 58.0 (16.91); 10/10 (20) China	TN	RCT, double blind	75 U, intradermal, submucosa, 15 injections	VAS AE
Xiao et al. (2010)	BTX‐A: 70.0 (15.4); 11/9 (20) Placebo: 67.0 (12.1); 9/11 (20) China	PN	RCT, double blind	50−200 U, subcutaneous, 8–20 injections	VAS AE
Yuan et al. (2009)	Total: 65.6 (9.2); 6/12 (18) Taiwan	DNP	RCT, double blind	50 U per each foot, intradermal, 12 injections	VAS AE
Zuniga et al. (2013)	BTX‐A: 64.5 (12.94); 9/11 (20) Placebo: 66.06 (14.16); 10/6 (16) Argentina	TN	RCT, double blind	50 U, intramuscular, 5 injections	VAS
Zhang et al. (2014)	BTX‐A (25 U): 58.16 (11.54); 10/15 (25) BTX‐A (75 U): 62.64 (13.32); 12/16 (28) Placebo: 58.41 (11.74); 14/13 (27) China	TN	RCT, double blind	25 U, 75 U, intradermal, submucosa, 20 injections	VAS

Abbreviations: AE, adverse event; DNP, diabetic neuropathic pain; F, female; M, male; NPS, neuropathy pain scale; NRS, numeric rating scale; PN, postherpetic neuralgia; PNP, peripheral neuropathic pain; TN, trigeminal neuralgia; VAS, visual analog scale.

### 3.2 Risk of bias

**Figure 2:**
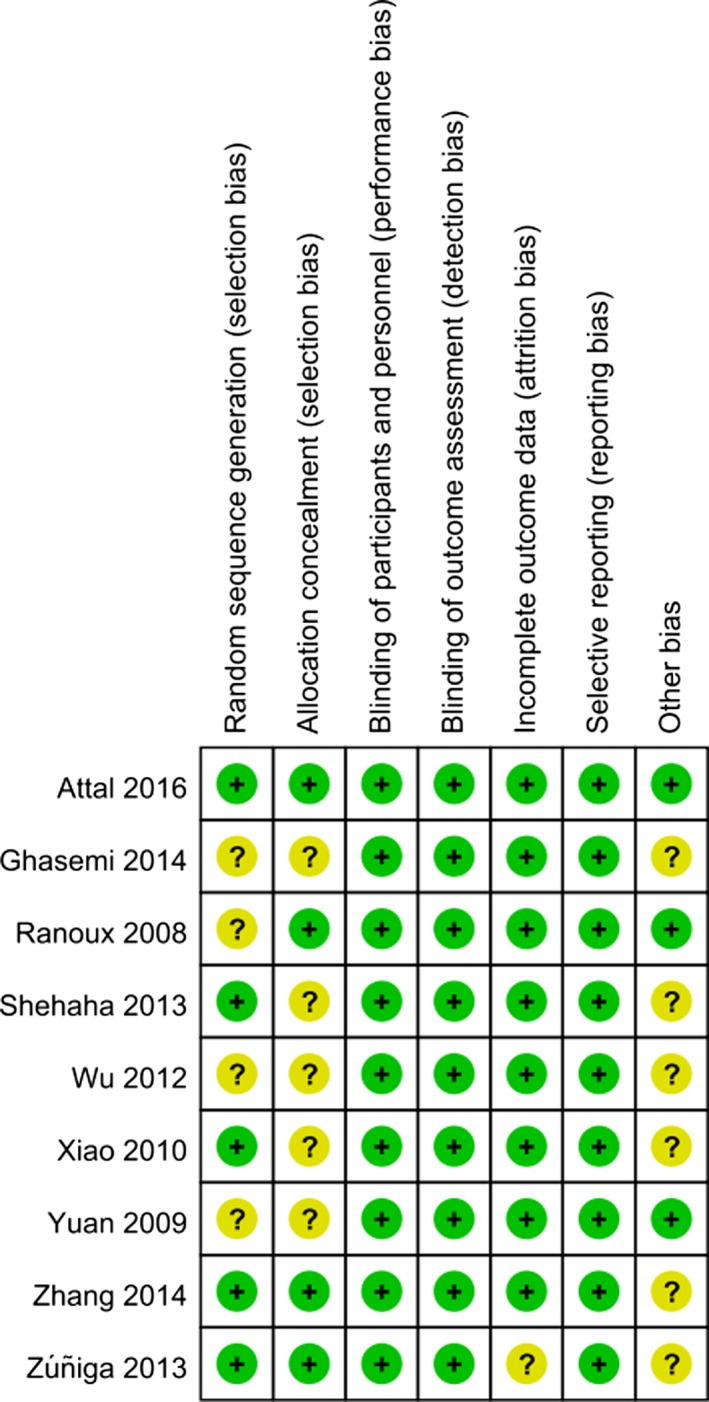
Cochrane bias assessment for individual trial

**Figure 3:**
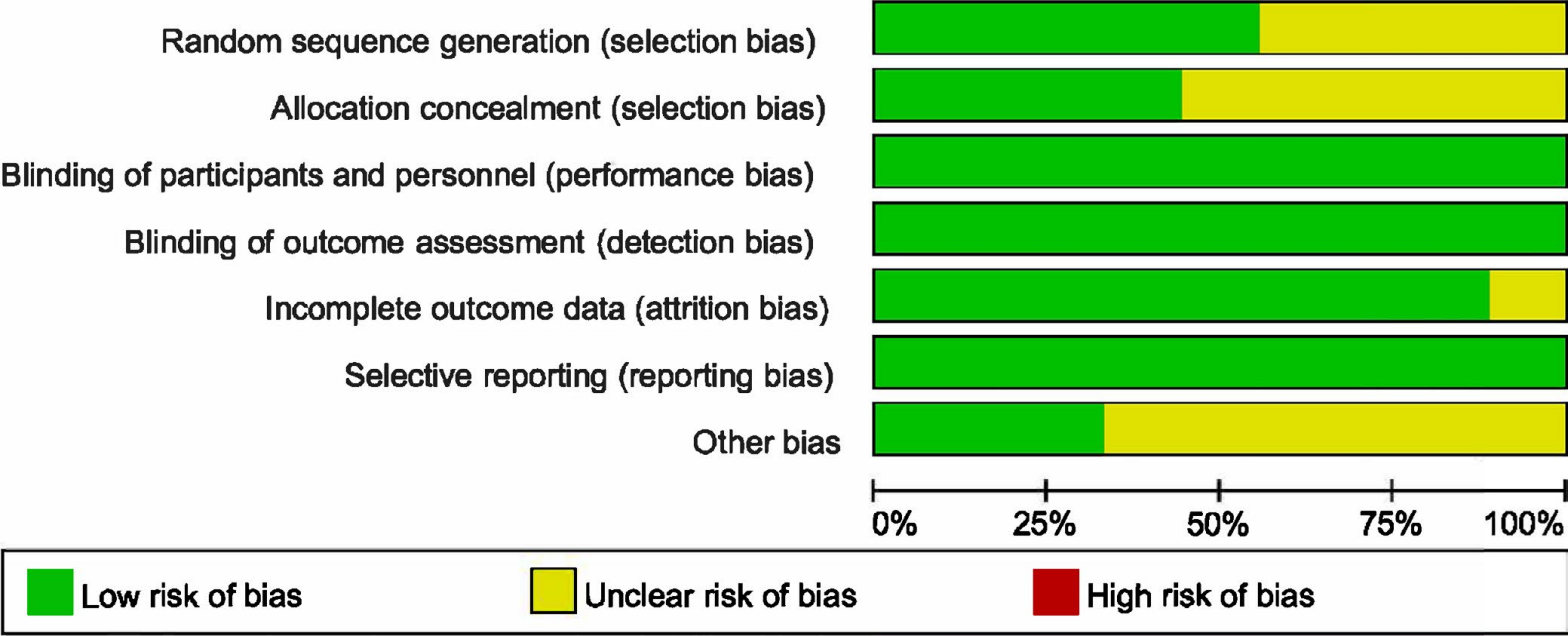
Graph of risk of bias for eligible RCTs

**Figure 4:**
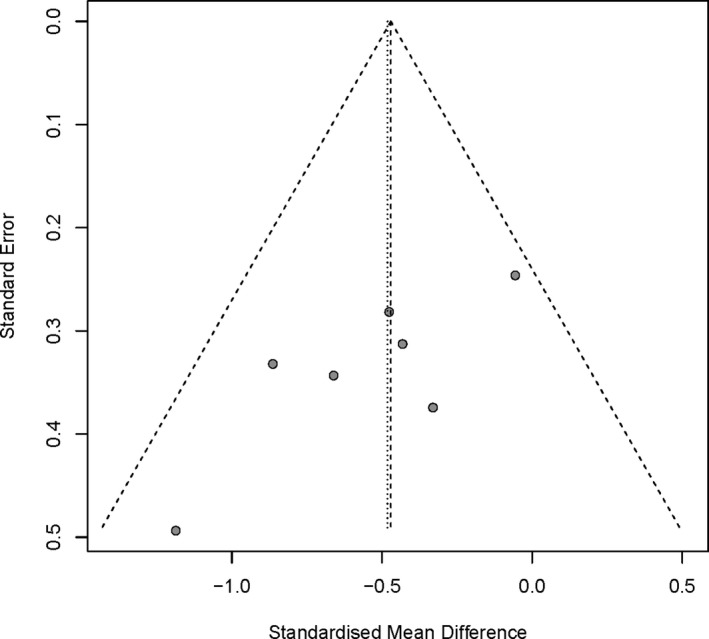
Bias assessment plot for the effect of BTX‐A on pain score by funnel blot

The quality of included trials is illustrated in Figure 2. Authors' evaluations on the risk of each biased item presented as percentage are shown in Figure 3. Assessment of publication bias was conducted based on funnel plot. There was no obvious publication bias on the grounds of almost symmetric funnel plot for the effects of BTX‐A on pain scale (Figure 4). However, the risk of bias across all included trials was medium due to unclear quality and potential publication bias.

### 3.3 Clinical outcomes

**Figure 5:**
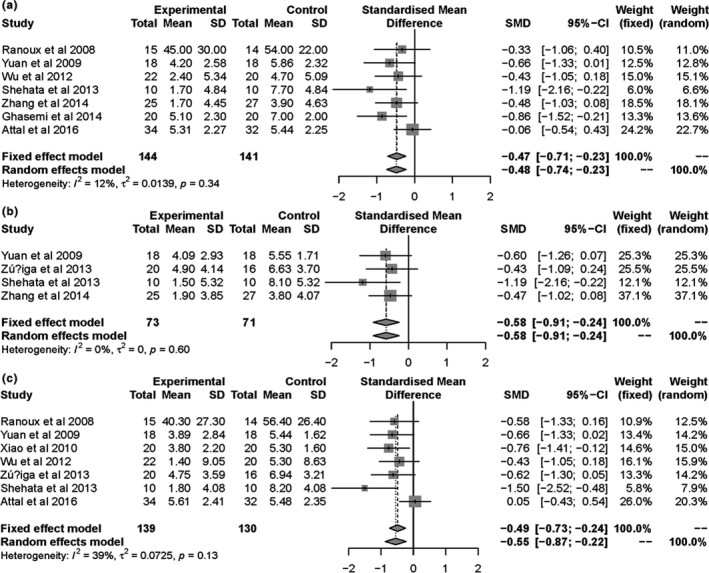
Forest plots of standard mean difference in pain score for BTX‐A versus placebo at 1 month (a), 2 months (b), and 3 months (c)

**Figure 6:**
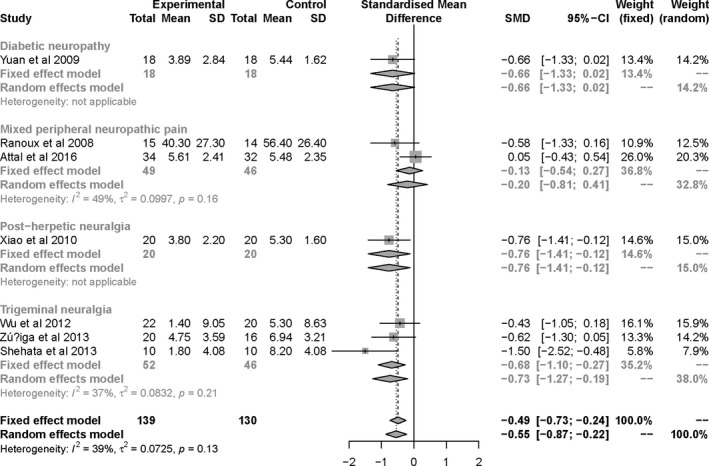
Forest plots of standard mean difference in pain score for BTX‐A versus placebo at 3 months, and subgroup analyses for different types of NP

**Figure 7:**
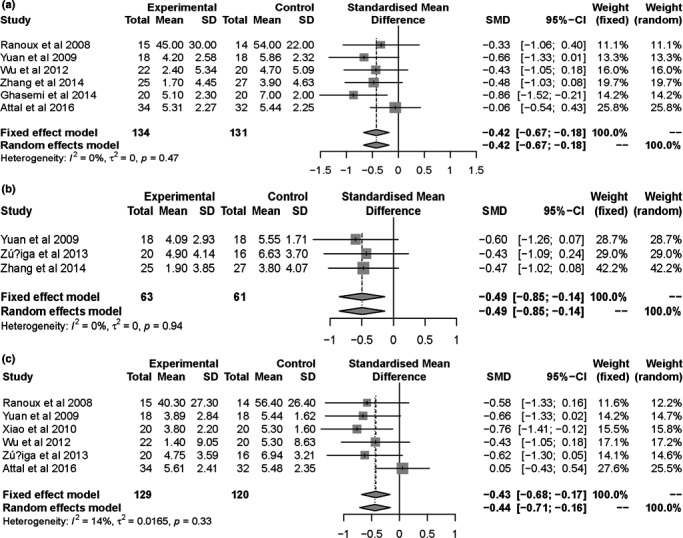
Forest plots of standard mean difference in pain score for BTX‐A versus placebo at 1 month (a), 2 months (b), and 3 months (c), excluding the single‐blind study (Shehata et al., 2013)

**Figure 8:**
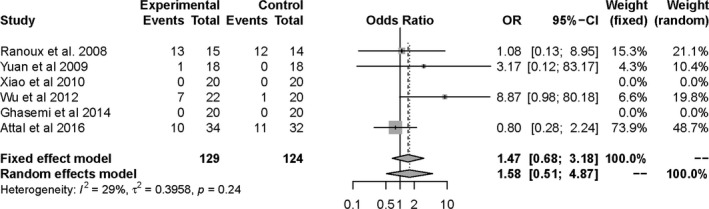
Forest plot of odds ratio (OR) in adverse events comparing BTX‐A with placebo

Patients' clinical outcomes from this review include: (a) mean visual analog scale (VAS) score, neuropathy pain scale (NPS), and numeric rating scale (NRS) at the end of follow‐up. The final analysis showed statistically significant reduction in pain score for BTX‐A group (SMD = −0.48, 95% CI [−0.74, −0.23] at the first month, SMD = −0.58, 95% CI [−0.91, −0.24] at the 2nd month, and SMD = −0.55, 95% CI [−0.87, −0.22] at the 3rd month; Figure 5). Furthermore, we conducted a subgroup analysis to assess which type of neuropathic pain benefited from the injections of BTX‐A (Figure 6). The subgroup results showed that the pain intensity of TN and PN was reduced more. There is no obvious bias on including and excluding the single‐blind study (Shehata et al., 2013; Figure 7). (b) NNT for respondents with pain score reduced >50% from baseline to endpoint. Based on NNT, the effect of BTX‐A is better for TN and PN than other PNP (Table 2). (c) Adverse events were related to BTX‐A injections. There was no significant difference in adverse events related to BTX‐A injections between BTX‐A and placebo (OR = 1.58, 95% CI [0.51, 4.87], *p* = .424), detecting no significant heterogeneity (*I* ^2^ = 29%; *p* = .24; Figure 8).

**Table 2:** OR and NNT for 50% pain intensity reduction of BTX‐A compared with placebo

Study	Diagnosis	EER (%)	Treated sample size	CER (%)	Placebo sample size	ARR	OR	NNT
Wu et al. (2012)	Trigeminal neuralgia	68.2	22	15	20	0.53	12.14	1.9
Apalla et al. (2013)	Postherpetic neuralgia	86.7	15	0	15	0.87	167.4	1.2
Ghasemi et al. (2014)	Diabetic neuropathy	30.0	20	0	20	0.3	18.38	3.3
Zhang et al. (2014)	Trigeminal neuralgia	70.4	27	32.10	28	0.38	5.01	2.6

Abbreviations: ARR, absolute risk reduction; CER, control event rate; EER, experimental event rate; NNT, number needed to treat; OR, odds ratios.

## 4 DISCUSSION

In the present meta‐analysis, our pooled data showed that BTX‐A provides significant benefit in the treatment of patients with TN and PNP compared with placebo, improving the proportion of respondents and pain scores at follow‐up. The reported adverse reactions were mild, transient, and nonsystemic. No serious adverse reactions were presented in the treatment than placebo arms in these studies. All these evidence suggested that BTX‐A is efficacious and safe in treating TN and PNP compared with placebo. However, the evidence was moderate owing to limited studies and small sample size.

As early as 2002, Micheli et al. reported that a 70‐year‐old man with hemifacial spasm associated with trigeminal neuralgia secondary to an ectatic basilar artery was treated with BTX‐A, and relief was gained not only from twitching but also from pain (Micheli, Scorticati, & Raina, 2002). In addition, in 2005, Allam et al. reported that BTX‐A injections elicited a stable analgesic response and long‐term pain control in a patient with intractable TN (Allam, Brasil‐Neto, Brown, & Tomaz, 2005). Subsequently, a randomized, open‐ended study was conducted by Türk et al. to evaluate the efficacy of botulinum injections in cases of refractory trigeminal neuralgia. They demonstrated that BTX‐A can be effective in cases of intractable trigeminal neuralgia (Turk, Ilhan, Alp, & Sur, 2005). Furthermore, thirteen patients with trigeminal neuralgia treated with BTX‐A were investigated by Piovesan et al. in an open‐label pilot study. After BTX‐A, VAS score, surface area of pain, and therapeutic coefficient were reduced in all patients and for all branch trigeminal nerves studied (Piovesan et al., 2005). These findings indicated that BTX‐A has a beneficial effect in the treatment of TN.

Recently, three reviews have demonstrated that BTX‐A may provide a clinically significant benefit in treating TN (Morra et al., 2016), PN (Shackleton et al., 2016), and DNP (Lakhan et al., 2015). However, thus far, few investigations have explored the evidence for the overall effect of BTX‐A on TN and PNP. Therefore, we conducted this meta‐analysis to fill this gap. Our review showed that the incidence of adverse events from BTX‐A was similar to placebo and was consistent with other studies (Lakhan et al., 2015; Morra et al., 2016). Nonetheless, our effect size is different from other reviews in this field. We chose SMD as effect size due to different measures from those trials. Our results showed that BTX‐A reduced SMD of pain score by −0.48 (−0.74, 0.23), −0.58 (−0.91, −0.24), and −0.55 (−0.87, −0.22), at 4, 8, and 12 weeks, respectively, compared with placebo. Furthermore, we conducted a subgroup analysis owing to heterogeneity at 12 weeks, indicating that BTX‐A reduced SMD of pain score by −0.76 (−1.41, −0.12) in PN and −0.73 (−1.27, −0.19) in TN, compared with placebo. Current meta‐analysis provides evidence that BTX‐A is superior to placebo in relieving TN and PNP, especially PN. Why is BTX‐A better for PN than other peripheral neuropathic pain? These reasons may interpret these discrepancies, including better effect of this drug on the mechanisms of pain paroxysms, and the use of different preparations of BTX, which are not bioequivalent (Ranoux, Gury, Fondarai, Mas, & Zuber, 2002). Non‐neuropathic pain and other neurological conditions were also involved in the complex mechanisms of PNP (Cohen & Mao, 2014). The mechanism of BTX acts on neuropathic pain involves deactivating the sodium channel and inhibiting the release of inflammatory mediators and peripheral neurotransmitters from sensory nerves (Park & Park, 2017). BTX‐A changes the Na current of a neuronal excitable membrane and controls the Na current with a non‐concentration‐dependent manner, which is different from tetrodotoxin, antiepileptic drugs, and local anesthetics (Cui, Khanijou, Rubino, & Aoki, 2004). The results of subgroup analysis also show good agreement with the findings from previous studies. However, there are sources of variability among studies including injection sites, recurrent injections, dosing regimens, baseline characteristics of subjects, and time of follow‐up. The small number of studies available for review, the diversity of treatment options, limited our ability to more accurately assess true treatment outcomes. So, the conclusion should be interpreted with cautious. This raises the need for future trials, with a focus on which dose of BTX‐A is optimal for pain relief, how long the duration of treatment lasts, and which route of administration is optimal.

To our knowledge, this is the first meta‐analysis to assess the overall impact of BTX‐A on TN and PNP compared with placebo. However, several limitations associated with this meta‐analysis should be noted. First, because of the limited number of studies included in this meta‐analysis, type‐II errors owing to chance cannot be completely excluded (Thorlund et al., 2011). In theory, randomization can eliminate the problem of unknown confounders, but the randomization of a small number of patients may lead to an imbalance in the arms. Therefore, our meta‐analysis may produce biased results, especially considering that meta‐analysis is based on the normality hypothesis of the significance test (Jackson, Kuriyama, & Hayashino, 2012; Lakhan et al., 2015). Second, meta‐analysis has been often criticized for the inclusion of poor‐quality trials and the potential of publication bias (Rosenthal & DiMatteo, 2001). Most studies characterized by small samples and poor methodological quality were included in this meta‐analysis. In addition, the risk of bias in most trials is unclear. Therefore, the possibility of biased results could not be ruled out. To avoid this debate, more well‐designed RCTs were required. In addition, an objective assessment of heterogeneity and publication bias also helps to maintain reliable conclusions. Third, our search strategy only included trials in English database and excluded trials of other languages, which may lead to, some extent, selective bias. The treatment protocols of those trials have some differences. We cannot conclude from the current limited trials which strategy should be the best approach.

## 5 CONCLUSIONS

In summary, our pooled results support the injection of BTX‐A as a promising alternative treatment for TN and PNP. Further larger and well‐designed RCTs are still needed to provide more in‐depth insight into current issues.

## CONFLICT OF INTEREST

The authors declare no conflict of interest.

## Data Availability

The data that support the findings of this study are available from the corresponding author upon reasonable request.
